# An Uncommon Case of Upper-Extremity Mucormycosis in a Patient With Acute Myeloid Leukemia

**DOI:** 10.1016/j.jhsg.2023.11.001

**Published:** 2023-11-29

**Authors:** Teren Yedikian, Ryu Yoshida, Meghan McCullough, Peter Deptula, David Kulber

**Affiliations:** ∗ Department of Hand and Upper Extremity Surgery, Cedars-Sinai Medical Center, Los Angeles, CA

**Keywords:** AML, Mucorales, Mucormycosis, Upper extremity, Zygomycetes

## Abstract

Upper-extremity mucormycosis is a rare, life-threatening fungal infection mainly affecting immunocompromised patients. We report a case of a 30-year-old woman with acute myelogenous leukemia who developed this infection during her hospital stay. The culprit was Mucorales, a subgroup of Zygomycetes species known for fast-progressing, highly lethal infections. She presented with fever, chills, and a lesion on her left forearm that worsened despite initial broad-spectrum antibiotics. A punch biopsy confirmed the diagnosis, leading to antifungal therapy with isavuconazonium sulfate and later amphotericin B, combined with surgery. Timely intervention is critical because delayed treatment can result in severe complications and death. Early suspicion, histology, microscopy, and fungal cultures are vital for accurate diagnosis. Treatment primarily involves amphotericin B, whereas adjunctive therapies such as topical amphotericin B and hyperbaric oxygen show promise. This case underscores the importance of prompt medical and surgical action, enhancing early detection of mucormycosis in immunocompromised patients.

Zygomycosis is a term used to describe fungal infections caused by Zygomycetes species that are classified into two orders, one of which is Mucorales. Fungi from this order cause mucormycoses, a spectrum of rapidly progressing infections with high mortalities, particularly among immunosuppressed patients.^1^ A recent meta-analysis of patients with mucormycosis reported that 33% of the patients have a concomitant hematological malignancy. Of that group, 42% were diagnosed with acute myeloid leukemia.^2^ Although these infections are not infrequent in this patient population, mucormycosis infections of the upper extremity in immunocompromised patients are rare. In a 12-year retrospective review at an academic institution, less than 1% of mucormycosis cases in the upper extremity were seen in immunocompromised individuals.^3^ Case reports of patients with hematological malignancy and upper-extremity mucormycosis presented after the induction of chemotherapy or in the setting of neutropenic fever.^4^ We present a case of a 30-year-old woman with acute myelogenous leukemia presenting with fever, nausea, and chills who developed an upper-extremity lesion during her hospital stay. The aim of the study was to describe a case of upper-extremity mucormycosis in an immunocompromised individual, its treatment, and clinical presentation. Written informed consent was obtained from the patient regarding the publishing of this report and associated figure images.

## Case Report

A 30-year-old woman with a medical history of acute myelogenous leukemia harboring normal cytogenetics and mutations of NPM1 and IDH2, who underwent 7 + 3 induction therapy with cytarabine and anthracycline followed by Enasidenib, is currently receiving high-dose cytarabine consolidations, presented to her hematologist and oncologist’s clinic with a 1-day history of fever, nausea, and chills, accompanied by brown urine for 3–4 days. She did not take any medications to alleviate her symptoms. She also reported that her children were ill but denied any recent travel. Three weeks earlier, she was admitted to the hospital for extended spectrum beta-lactamase *Escherichia coli* bacteremia, which resolved after a 2-week course of meropenem. One week before this clinic visit, she was treated for a urinary tract infection caused by *Enterococcus faecalis* with a 5-day course of nitrofurantoin. In the clinic, she was febrile to 103.8˚F and tachycardic in the 120s. Bloodwork was significant for a white blood cell count of <30/UL; hence, the patient was sent to the emergency room to be admitted for neutropenic fever and broad-spectrum antibiotic treatment.

**Figure 1:**
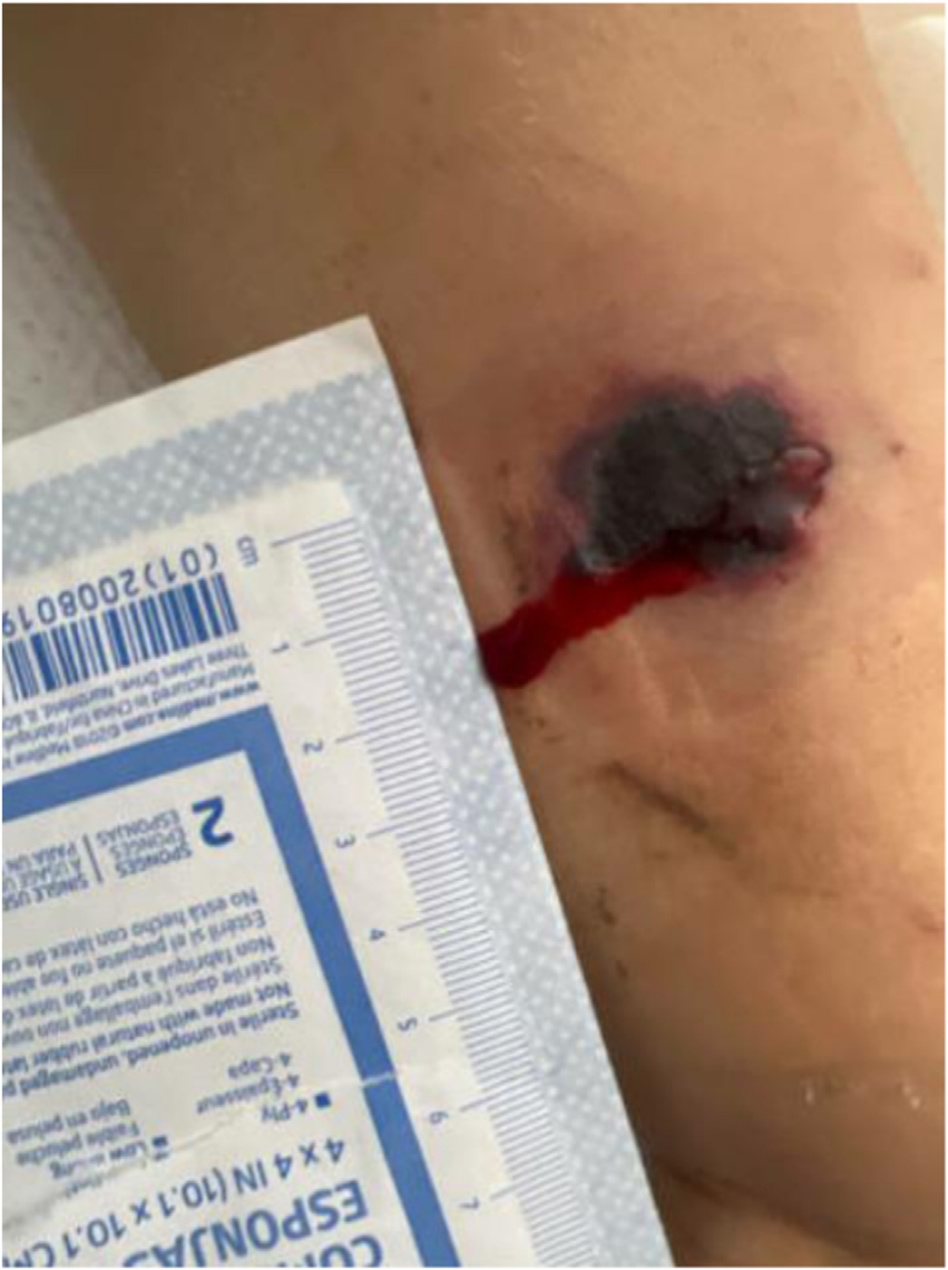
2 × 2 cm necrotic, dusky lesion of left volar forearm with surrounding erythema.

She was placed on IV vancomycin and meropenem and was prophylactically placed on atovaquone, acyclovir, and posaconazole. After 7 days of treatment, she reported pain and red spots underneath adhesive tape used to hold down her IV catheter on her left volar forearm. Due to concurrent left arm numbness, an upper-extremity venous ultrasound was ordered to rule out a deep vein thrombosis. The preliminary result the following day was negative; hence, the violaceous nature of the lesion was attributed to extravasation from thrombocytopenia. After an additional day passed, the rash had progressed to a lesion that was necrotic, centrally dusky with surrounding erythema and edema (Fig. 1).

**Figure 2:**
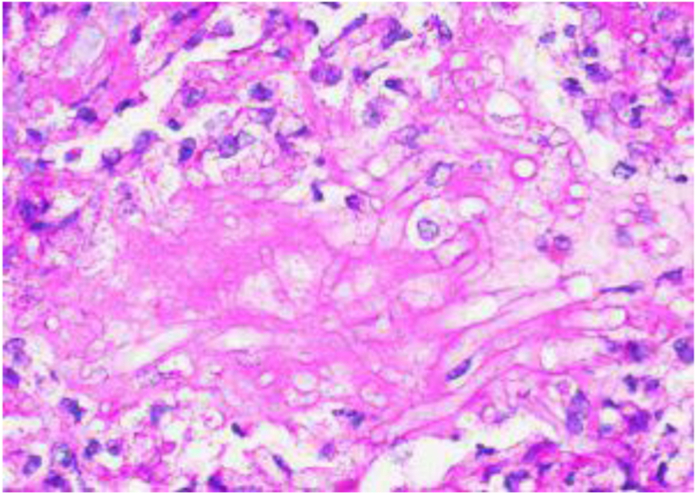
PAS 60× magnification of left volar forearm lesion.

**Figure 3:**
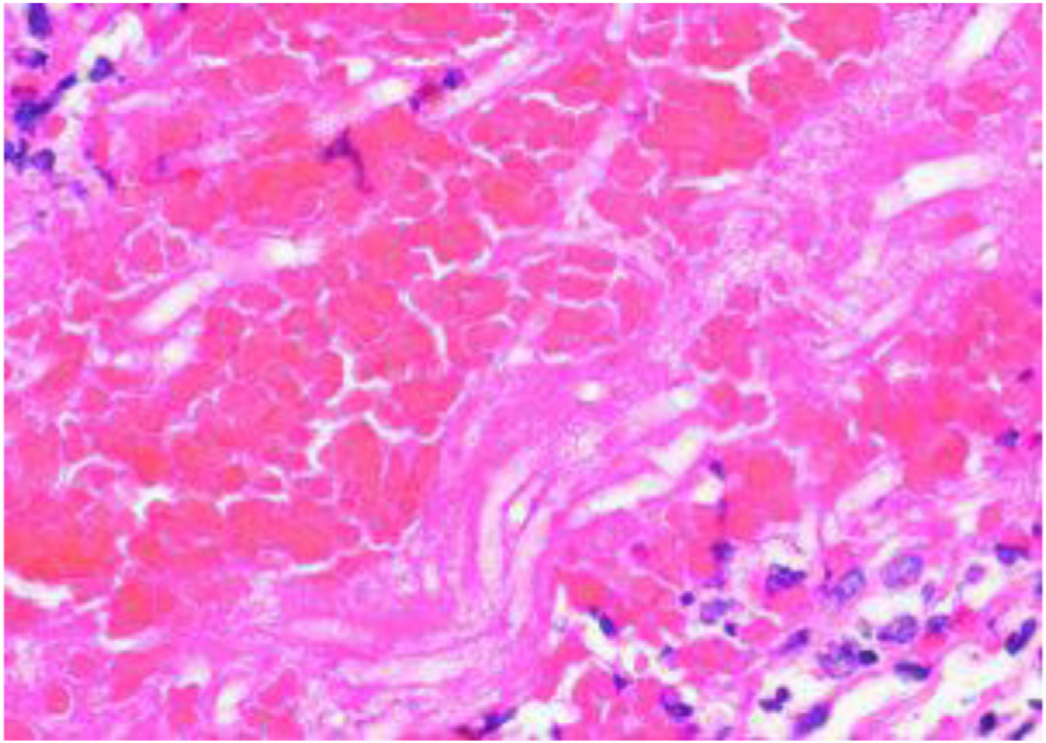
H&E 60× magnification of left volar forearm lesion.

The dermatology team performed a punch biopsy 2 days after the initial lesion presentation with fungal cultures, and she was started on isavuconazonium sulfate due to suspicion of a fungal infection. The biopsy resulted 2 days after with the organism morphology being consistent with Mucorales/Zygomycetes group. Microscopic findings on Periodic acid–Schiff (PAS) and hematoxylin and eosin (H&E) staining showed deep dense, vasculocentric, and necrotizing inflammation and fibrin thrombi with the presence of branching fungal hyphae within walls and lumens of vessels (Figure 2, Figure 3).

**Figure 4:**
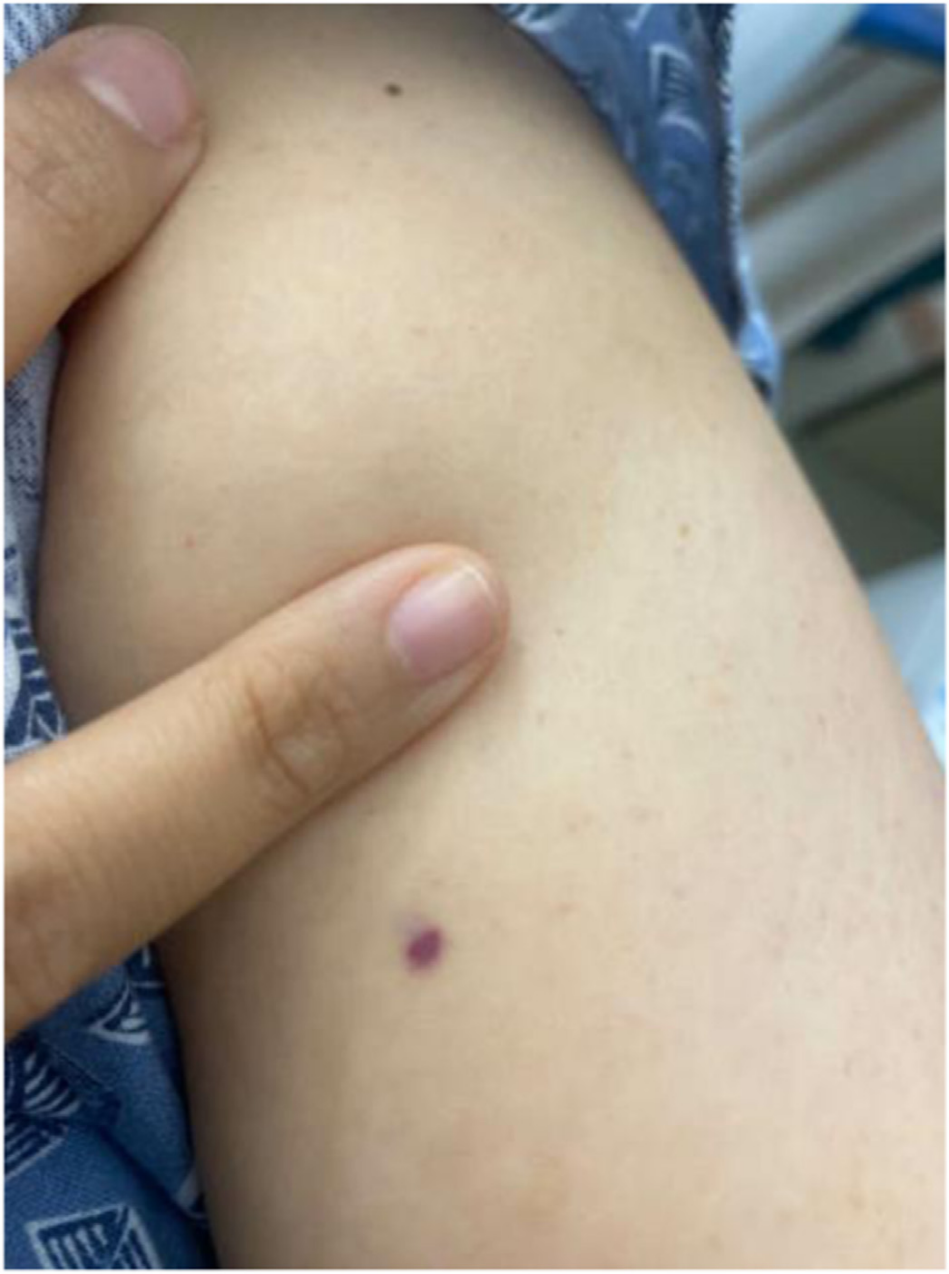
3 × 3 mm violaceous lesion of left volar arm.

Once the inciting organism was identified, she received a central line. Isavuconazonium sulfate was stopped, and she was started on amphotericin B. Hand surgery was consulted for surgical intervention the same day. They noticed a small lesion on her left upper arm (Fig. 4) and planned to surgically excise that lesion along with the lesion on her left volar forearm. An 8 × 8 cm area of skin, subcutaneous tissue, and fascia were excised, irrigated, and debrided from her left volar forearm and a 1 × 1.5 cm area of skin, and subcutaneous tissue was excised from her left upper arm. The left upper arm lesion was sent for culture and was negative for any organism growth. The defect was closed primarily with sutures.

She underwent excision of both lesions and irrigation and debridement of the left volar forearm lesion. The left upper arm lesion was sent for culture and was negative for any organism growth.

After her procedure, computerized tomography scans of her maxillofacial, sinuses, brain, and chest revealed no acute indications of sinopulmonary mucormycosis, although some trace mucosal thickening of the right maxillary sinus exists. The following day, the patient reported pain behind her right eye, ear, and along the right side of her throat. The otolaryngology team was consulted; however, no recommendations were made since her symptoms resolved uneventfully over the following days. She remained on amphotericin B for 5 days, and she was discharged on isavuconazonium sulfate until follow-up with an infectious disease physician. Her incisions healed uneventfully over the next few weeks.

## Discussion

We present a rare case of upper-extremity mucormycosis in an immunocompromised host. As seen in this case, mucormcycosis infections in immunocompromised patients can occur around areas of IV placement and can quickly progress in severity.^5^ Clinicians need to be equipped with a high index of suspicion because the most important factor for mucormycosis management is early diagnosis. Although variable, the common clinical presentation of upper-extremity mucormycosis infection is a necrotic lesion with surrounding erythema.^5^ Cutaneous lesions are the second most common manifestation of mucormycosis, which are preceded by rhino-orbital-cerebral infections and followed by pulmonary mucormycosis.^2^ Prolonged infection may lead to amputation and mortality of the upper extremity.^4^ Mortality remains high in both those with cutaneous disease (31%) and disseminated disease (68%).^2^ Dissemination presents as an infection in two or more sites or the presence of mucormycosis in the bloodstream. Concerns about dissemination in this patient resulted in the second lesion in her arm to be excised and examined.

The current gold standard for identifying mucormycosis infection is histological and microscopic observation along with fungal cultures.^6^ A 10% potassium hydroxide stain can help visualize broad, nonsepatate hyphae with 90° branches. However, PAS and H&E staining can demonstrate vascular invasion and thrombosis of small vessels that help confirm the diagnosis.^5^ Newer advancements in technology shine light on the use of polymerase chain reaction to detect fungal DNA; however, laboratory standards are still in development.^6^

Treatment for mucormycoses infections relies primarily on antifungal therapy with amphotericin B, followed by posaconazole and isavuconazole for stepdown or salvage therapy once clinical response has been achieved with amphotericin B.^7^ Combination antifungal therapy is used by some physicians; however, no clinical trials demonstrate that combination therapy had a greater efficacy over amphotericn B alone. In addition to antifungal treatment, surgical debridement has been shown to increase patient survival and should be performed in a timely manner.^1^ Similar guidelines have been agreed upon as part of the “One World, One Guideline” initiative of the European Confederation of Medical Mycology.^8^

Adjunct treatment has also been reported in the literature. In one case report, a patient with disseminated disease was given topical amphotericin B 3% ointment in a mineral oil-hydrophil petrolatum base in addition to their treatment regimen of oral amphotericin B and isavuconazole.^9^ This patient was able to eradicate the skin lesions without surgical intervention. Hyperbaric oxygen has been used as adjunctive therapy and is theorized to increase free oxygen radicals, increase neutrophil phagocytosis, and improve angiogenesis.^10^ Although both treatments have demonstrated success, further studies need to be conducted to determine their use with the current gold standard of treatment.
